# Influence of Berry Heterogeneity on Phenolics and Antioxidant Activity of Grapes and Wines: A Primary Study of the New Winegrape Cultivar Meili (*Vitis vinifera* L.)

**DOI:** 10.1371/journal.pone.0151276

**Published:** 2016-03-14

**Authors:** Xu Liu, Jinlu Li, Yuping Tian, Mingan Liao, Zhenwen Zhang

**Affiliations:** 1 College of Enology, Northwest A&F University, Yangling, Shaanxi, China; 2 Shaanxi Engineering Research Center for Viti-Viniculture, Yangling, Shaanxi, China; 3 Department of Horticulture, Sichuan Agricultural University, Yaan Sichuan, China; UMR INSERM U866, FRANCE

## Abstract

Wine grapes are usually harvested in vineyards when they ripen. However, not all of the berries in a vineyard ripen homogeneously because of different microclimates around the clusters and berries. In this study, the influence of berry heterogeneity on the phenolic content and antioxidant capacity of grapes and wines under a continental monsoon climate was evaluated for a new wine grape cultivar Meili (*Vitis vinifera* L.). The total phenolic, flavonoid, flavanol, and monomeric anthocyanin contents in the skin and wine significantly increased with grape density; however, there was no significant difference in the seeds between the two lower densities. The highest values of DPPH free radical-scavenging activity, cupric-reducing antioxidant capacity, and hydroxyl radical-scavenging activity in the skin, seed and wine were detected for the densest berries. The sum of individual phenolic compounds in skin, seed and wine increased with berry density, though no significant difference for skin was observed between the two higher density classes. Hence, the chemical components of Meili grapes and wines were positively associated with the berry density at harvest under the continental monsoon climate.

## Introduction

The quality of red wine is highly associated with the degree of grape maturity at harvest [1]. The phenolic compounds in grape skin and seed perform important functions for high-quality red wine and contribute to a wine’s organoleptic characteristics [2]. Phenolic compounds also contribute to multiple biological effects, such as the antioxidant activity of grapes and wine [3,4].

Although grapes are usually harvested at the ripening stage, not all of the berries in a vineyard ripen homogeneously because of different microclimates around the clusters and berries [5]. Indeed, the biological characteristics of all berries from one vineyard are highly heterogeneous at any given time [6]. The variability in polyphenol concentration in grapes depends on the grapevine variety and is also influenced by viticultural practices and environmental factors [7,8]. The location of vines in a vineyard, the cluster position on the vine, berry orientation in a cluster, and even the berry size may induce obvious differences in physicochemical characteristics among berries [9–11]. Considering that one grape cluster consisting of more than one hundred berries develops from a single panicle, differences among the outer and inner berries in the same cluster are notable [12].

Previous studies have generally provided the average value of sampling berries though have often overlooked berry heterogeneity [13,14]. A recent study reported the influence of grape heterogeneity at harvest on the physicochemical properties of berries and wine quality [15], and significant differences have been observed in the grape textural properties and anthocyanin contents of Cabernet Franc grapes of different density classes [16]. In addition, Rolle et al. found that the densimetric sorting of Nebbiolo grapes could be used to separate grapes with different quality parameters [17], and Italia table grape berries showed high variability over several classes of density distributions, even though 85% of the berries were classified into three density levels at harvest [18].

Most wine regions in the world benefit from a Mediterranean climate without heavy rainfall during grape ripening and soil coverage in winter. However, the continental monsoon climate characterized by frequent rain in combination with high temperature is common in the wine regions of China. Correspondingly, berries might grow more rapidly and be situated closer in a cluster following veraison. Inner berries may experience different microclimates, particularly less solar radiation and lower temperature. These environmental factors could influence berry development, phenolic compound accumulation, and the expression of flavonoid pathway genes [11,19]. Moreover, grape heterogeneity at harvest may exert more significant effects on berry and wine quality under the continental monsoon climate.

This study aimed to determine the variance of grape and wine composition among the three density classes of Meili berries. The objective of this work was to evaluate the influence of grape heterogeneity at harvest on phenolics profile and antioxidant activity of skin, seed, and wine. The results were primarily expected to provide fundamental information to viticulturists and winemakers for optimizing vineyard practices and winemaking management in a continental monsoon climate. Meili (*Vitis vinifera* L.) is a new wine grape cultivar with high resistance to downy mildew. It was selected through cross breeding since 1982 and was firstly registered on the Fruit Industry Bureau of Shaanxi Province in 2011.

## Materials and Methods

### Grape samples

Meili grapes were collected from the National Seedling Breeding Center of Grape at Yangling, Shaanxi Province in 2013. This breeding center belongs to private vineyard. No specific permissions were required for the experimental locations. The field studies did not involve endangered or protected species. The GPS coordinates of the experimental site is 34°30′ N, 108°29′ E. The main physicochemical properties of soil in vineyard include: loessial soil with a 8.34 of pH, 27% of field water capacity, 11.33 g kg^-1^ of organic matter, 44.5 mg kg^-1^ of available nitrogen, 7.8 mg kg^-1^ of available phosphorus, 120.4 mg kg^-1^ of available potassium. All grapevines had been planted for 6 years with own-rooted seedlings and trained to a vertical trellis with Guyot pruning. The south–north oriented rows were arranged with 2.5 m spacing between rows and 1.0 m spacing between vines. The average grape yield per vine was close to 4 kg. The vineyard was routinely managed using standard fertilizer supply program and integrated disease and pest management (IDPM). The basic climate parameters in 2013 were: average temperature 13.2°C, relative humidity 70.9%, rainfall 627.2 mm, sunshine hours 1911. The grape samples were harvested at August 26, the time of optimal maturity for Meili wine production. The average level of vineyard ripeness was determined as reducing sugar 171.25 g L^-1^, total acidity (tartaric acid) 8.62 g L^-1^ and pH 3.01. A sample of approximate 30 kg berries with pedicels attached was randomly collected from the center rows of a plot by picking bunches of 3–5 berries from each cluster. Three samples were collected as replicates. For each sample, the berries were detached from stalks using a scissors and then sorted according to density, which was estimated by flotation in different saline solutions (100–170 g L^-1^ sodium chloride) [20]. These solutions had densities between 1064 and 1103 kg m^-3^. Finally, five density classes were recorded: D1 = 1064 kg m^-3^, D2 = 1071 kg m^-3^, D3 = 1076 kg m^-3^, D4 = 1082 kg m^-3^, D5 = 1089 kg m^-3^. The percentage of D1 and D2 berries from total sample was only 1.03% and 4.94%, respectively. Most of the berries belonged to class D3, D4, and D5. The floating berries at these three densities were separated and washed with distilled water. For each density, one subsample of 30 berries was randomly chosen for measurement of physical properties. Three subsamples of 200 berries were used to assess the phenolic profile and antioxidant capacity.

### Winemaking

For each density class, the grapes remaining after chemical analysis were crushed by hand and then transferred to 30 L stainless-steel containers according to the production process for red wine. The Meili wines of each density were produced in three replications. The must was treated with 60 mg L^-1^ of sulfur dioxide and inoculated with 20 g hL^-1^ of active dry yeast (*Saccharomyces cerevisiae* RC 212, Lallemand, Danstar Ferment AG, Switzerland), which was used to initiate fermentation according to commercial specifications. The fermentation lasted for 7–8 days at 25–28°C. The sugar consumption and temperature were monitored daily during fermentation, and the tank content was homogenized every day to dissolve the cap into the wine. The wines were then decanted to another tank, exposed to cold treatment for 3 weeks at 4°C, and then bottled.

### Physical and technological maturity parameters

Reducing sugars, pH, and total acidity of berries were determined according to O.I.V methods [21]. The weight was measured individually for 30 berries. Afterward, the distance between the top and bottom of each berry and the diameter of the equator were measured. The surface area and volume were calculated according to the method of Río Segade et al. [22].

### General enological parameters

The general parameters of wines including alcoholicity, residual sugars, pH and titratable acidity were analyzed according to the official methods established by O.I.V [21].

### Extraction of phenolics from skin and seed

The extraction of phenolic compounds was mainly based on the proposal of Meng [23]. For each density class, the fresh skins of the collected grapes were ground with liquid nitrogen. The ground powder (3.000 g) was extracted with 30 mL of acidified methanol solution (0.1% HCl, 60% methanol) at 25°C for 30 min in an external water bath with ultrasonic assistance. The extracts were centrifuged at 8000×*g* for 15 min at 4°C using a Sorvall RC-5C Plus centrifuge (Kendro Laboratory Products, Newton, CT, USA). The extraction procedure was repeated four times under identical conditions. All supernatants were combined and stored at –20°C in the dark. Then, 1.500 g of seed powder ground in liquid nitrogen was extracted three times using the same procedure. All of the supernatants were combined and kept at –20°C until use.

For HPLC analysis of individual phenolic compounds, 50 mL of sample (skin or seed extract or wine) was transferred to a 250 mL evaporation flask and concentrated to a volume of 10 mL in a rotary evaporator (SENCO-R series, Shanghai Shensheng Biotech, Shanghai, China) at 35°C. The aqueous phase was then extracted three times with 10 mL of ethyl acetate. Thereafter, the organic phases were combined and evaporated to dryness under vacuum at 35°C. The dried residuals were dissolved in 4 mL of methanol, filtered through a 0.45 μm filter, and analyzed by HPLC [24].

### Determination of phenolic content

#### Total phenolic content (TPC)

TPC of samples were measured by the Folin-Ciocalteu method [25]. Distilled water (5.99 mL), skin extract or wine (0.1 mL), and Folin-Ciocalteu reagent (0.2 mL) were placed in a test tube in sequence. Sodium carbonate solution (2 mL, 10%) was then added to the tube after reaction for 5 min. The mixture was kept from light and allowed to react at room temperature for 120 min, and the absorbance was measured at 765 nm. The results are expressed as mg gallic acid equivalent (GAE) g^-1^ skin or mg GAE L^-1^ wine. The seed extract was firstly diluted 5-fold with distilled water. Afterward, 100 μL of the diluted solution was transferred to a test tube for TPC determination using the same procedure used for the skin extract. The results are expressed as mg GAE g^-1^ seed.

#### Total flavonoid content (TFOC)

TFOC was determined according to the method of Jia et al. [26] with minor modifications. A 0.3 mL sample (skin or seed extract or wine) was mixed with 2.7 mL of methanol solution, 0.2 mL of sodium nitrite (0.5 mol L^-1^), and 0.2 mL of aluminum chloride (0.3 mol L^-1^) in sequence in a 10 mL centrifuge tube. After 5 min, 1.0 mL of sodium hydroxide (1 mol L^-1^) was added. The absorbance of the solution was measured against a blank at 510 nm. TFOC was assessed based on a calibration curve developed using rutin. The results are expressed as mg rutin equivalent (RE) g^-1^ skin or seed or mg RE L^-1^ wine.

#### Total flavanol content (TFAC)

TFAC was detected with *p*-DMACA [27]. Skin and seed extracts were diluted 2- and 5-fold, respectively, with distilled water. Then, 0.1 mL of the sample (the diluted solutions or wine) and 3.0 mL of *p*-DMACA solution (0.1% in 1 mol L^-1^ HCl in MeOH) were mixed and incubated at room temperature for 10 min. Absorbance was measured at 640 nm against a blank prepared following the same procedure as above but without the addition of *p*-DMACA. The results are expressed as mg (+)-catechin equivalents (CE) g^-1^ skin or seed or mg CE L^-1^ wine. TFAC was calculated from a calibration curve using (+)-catechin as the standard.

#### Total monomeric anthocyanin content (TMAC)

TMAC was determined using the pH differential method [28]. Anthocyanins are only synthesized in skin for red grapes and would be released into wine during maceration and fermentation. Skin extract and wine were diluted 20-fold with buffer at pH 1.0 and 4.5, respectively. Absorbance was measured at 510 and 700 nm in both pH 1.0 and 4.5 buffers and then calculated according to the equation A = (A_510_ –A_700_)_pH1.0_ –(A_510_ –A_700_)_pH4.5_. TMAC of the skin (expressed as cyanidin-3-glucoside equivalent, C3GE) was calculated using the formula TMA content (mg C3GE g^-1^ skin) = (A × MW × DF× V_e_ × 1000)/(ε × 1 × M), where A is the absorbance, MW is the molecular weight of cyanidin-3-glucoside (449 g mol^-1^), DF is the dilution factor, V_e_ is the extract volume, ε is molar extinction coefficient of cyanidin-3-glucoside (29,600), and M is skin weight. TMAC of wine was calculated according to the formula: TMA content (mg C3GE L^-1^) = (A × MW × DF × 1000)/(ε × 1), where the parameters are the same as for the former formula.

### Antioxidant activity determination

#### DPPH free radical-scavenging activity (DPPH)

DPPH was estimated by the method of Brand-Williams et al. [29]. Skin or seed extract or wine was mixed with DPPH methanolic solution and kept from light for 30 min. Absorbance was measured at 517 nm. The results are expressed as μM trolox equivalents (TE) g^-1^ skin or seed or μM TE L^-1^ wine.

#### Cupric-reducing antioxidant capacity (CUPRAC)

CUPRAC was determined according to the method of Apak et al. [30]. Briefly, skin extracts were diluted 5-fold with distilled water, whereas seed extracts or wine samples were diluted 10-fold with distilled water. The dilutions were then mixed with CuSO_4_, neocuproine, and distilled water. Absorbance was determined at 450 nm after 30 min. The results are expressed as μM TE g^-1^ skin or seed or μM TE L^-1^ wine.

#### Hydroxyl radical-scavenging activity (HRSA)

HRSA was measured using the procedure of Sroka & Cisowski [31], with minor modifications. Ferrous sulfate (100 μL, 0.02 mol L^-1^), 45 μL of hydrogen peroxide (0.15%), and 1 mL of salicylic acid (8 mmol L^-1^) were mixed in a test tube with 4 mL of distilled water. Then, 1 mL of sample (skin or seed extracts or wine) was transferred to the tube. The mixture was incubated for 30 min at 37°C, and the absorbance of the mixture was measured at 593 nm. The percentage of free radical-scavenging activity was calculated according to the equation: Scavenging effect (%) = [1–(A_sample_/A_control_)]×100.

### HPLC analysis for individual phenolic compounds

HPLC analysis was performed using a Shimadzu HPLC system with a Hibar RT LiChrospher RP-C18 column (250 × 40 mm, 5 μm), as based on the method of Cheng [24].

Solvent A and B were composed of 2% (v/v) acetic acid in water and acetonitrile, respectively. The following gradient was optimized after preliminary experiments: 0–15 min, 3%–6% B; 15–35 min, 6%–15% B; 35–55 min, 15%–30% B; 55–65 min, 30%–30% B; 65–80 min, 30%–0% B. The column temperature was 30°C, and the flow rate was 1.0 mL min^-1^. The PDA detector scanned from 200 to 400 nm. The analysis for each sample was performed in triplicate.

Phenolic compound identification was based on a comparison of their retention time with those obtained from authentic standards and also by spiking the samples with the standard solutions. Quantification was performed using the external standard method. A six-point calibration curve was used for each standard. All individual phenolic standards were purchased from Sigma-Aldrich Company (MO, USA).

### Statistical analysis

All data are expressed as the mean ± standard deviation (SD) and analyzed by one-way ANOVA. The means were separated by Fisher’s least significant difference (LSD) test using the SPSS package (version 19.0). Differences obtained at the *P*≤0.05 level were considered significant.

## Results and Discussion

### Grape distributions at harvest

**Fig 1:**
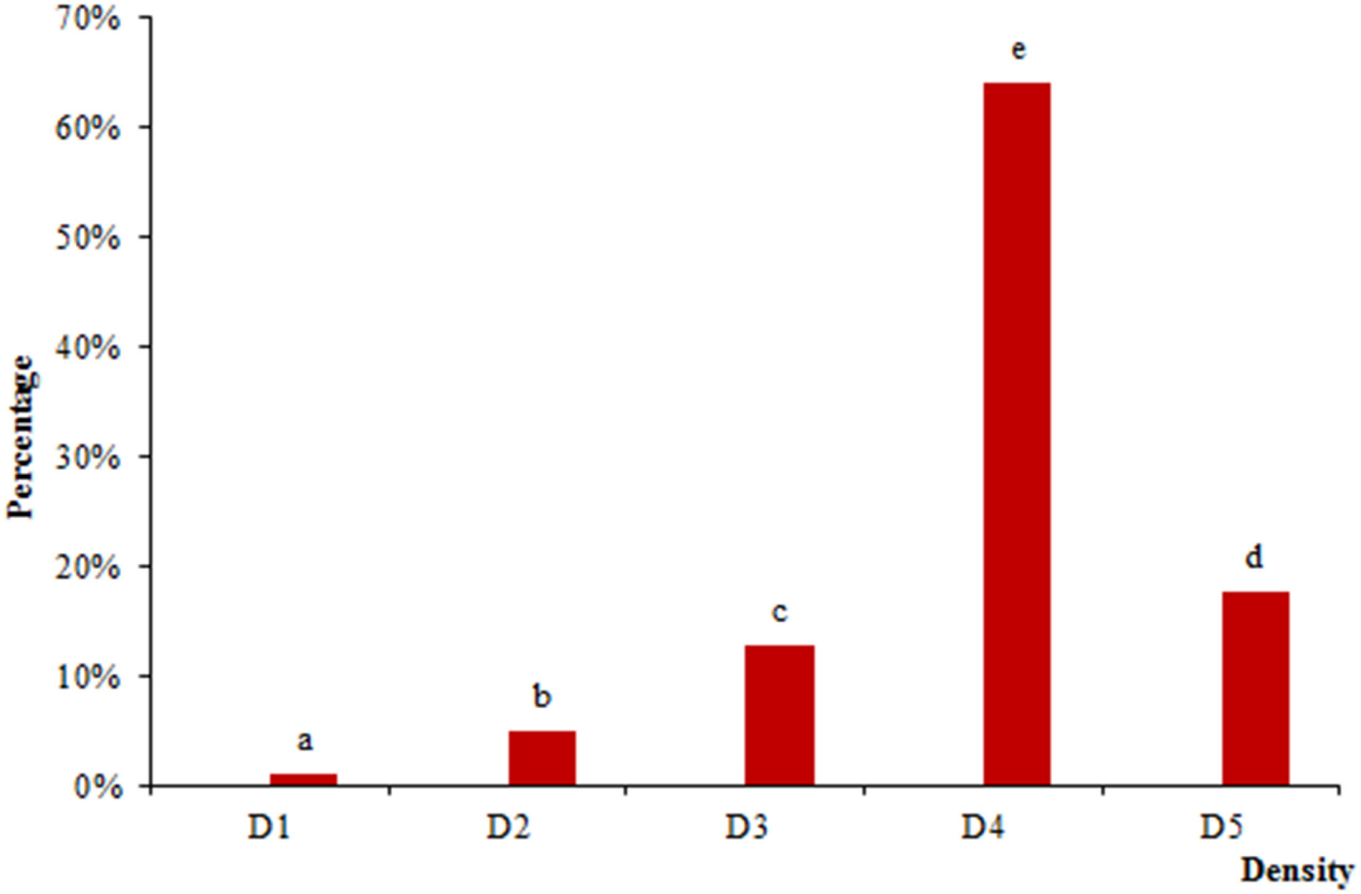
Percentage of Meili grape berries in different density classes.

As shown in Fig 1, variable percentages ranging from 1.03%–63.91% indicated berry heterogeneity for Meili grapes in different density classes at harvest. Up to 94.03% of the berries were distributed in density levels D3, D4, and D5. In particular, the D4 level had the highest percentage (63.91%) of berries. This heterogeneity of Meili grapes was less obvious than that observed in other wine grape varieties with wider percentage allocation [15,16]. Grapes on the vine usually suffer from different microenvironments which could result in a lack of homogeneous ripening in a vineyard, even within the same cluster [32]. The distribution of berries by density has occurred at veraison and changes during the ripening process [15]. Insufficiently ripened grapes are generally characterized by a low sugar content, high acidity, few anthocyanins, and high amounts of seed proanthocyanidins, which could adversely affect wine quality [1].

### Physical and technological maturity parameters

No significant difference was observed for berry weight, volume, and surface area of Meili grapes of the three densities (Table 1). However, the individual berry of Meili grapes outweighed that of Nebbiolo [15] and Carbernet Franc [16] grapes at the same soluble sugars level. The berry weight of Meili grapes ranged from 2.83–3.06 g per berry, significantly higher than the prominent wine grape cultivars, which are usually approximately 1.0–2.0 g per berry. Accordingly, Meili grapes are sometimes also used locally as table grapes. The reducing sugar contents increased markedly with grape density, up to a maximum in density D5 as presented in Table 1. The densest berries (D5) exhibited the lowest total acid content and highest pH value, though no significant differences were found between D3 and D4 level. Variance in sugar content of different berries were usually observed in one vine or cluster and may be attributed to different sunlight exposure. More photosynthate and higher activities of sugar synthase would be produced under higher solar radiation [32,33]. Furthermore, the densest berries visually showed the deepest skin color. These berries are assumed to be located around the exterior of a cluster, and they may receive more sunlight exposure. Hence, more anthocyanins may be synthesized and accumulated in the skin.

**Table 1:** Physical and technological maturity parameters of Meili grapes at harvest based on berry density classes.

Density level	Berry weight(g)	Volume(cm^3^)	Surface(cm^2^)	Reducing sugars(g L^-1^)	Total acidity(tartaric acid, g L^-1^)	pH
D3	3.06±0.60(a)^α^	2.46±0.44(a)	8.77±1.05(a)	152.33±2.08(a)	8.71±0.50(b)	2.98±0.03(a)
D4	2.92±0.54(a)	2.41±0.47(a)	8.66±1.15(a)	166.33±2.52(b)	8.58±0.55(b)	3.02±0.05(a)
D5	2.83±0.51(a)	2.29±0.40(a)	8.38±0.98(a)	182.67±2.08(c)	7.32±0.66(a)	3.15±0.07(b)

^α^ Values are the mean ± standard deviation (n = 30 for berry weight, volume, and surface; n = 3 for reducing sugars, total acidity, and pH). Within columns, significant differences according to the Fisher’s LSD test at the *P* ≤ 0.05 level are indicated by different letters.

### The wine parameters

The alcohol content of Meili wines increased with grape density (Table 2). However, the alcohol content was a slightly low; in particular, the wine made from grapes at the D3 level was only 8.99% (v/v). This was related to lower sugar content in grapes of density D3 as presented in Table 1. Extra attention should be paid on Meili grapes production in a continental monsoon climate because the rainfall during grape ripening, which largely contributed to the total annual precipitation was usually measured and would impede sugar accumulation in grape berries to some extent. When suffering from heavy rainfall during ripening, Meili grapes even need to be picked ahead of the normal harvest schedule. The total titratable acidity decreased with grape density. However, no significant difference was determined among the three density levels.

**Table 2:** Wine parameters.

Wine sample	Alcohol content(%,V/V)	Titratable acidity (tartaric acid, g L^-1^)	pH	Residual sugars(g L^-1^)
D3^α^	8.99±0.90(a)^β^	6.48±0.33(a)	3.02±0.22(a)	1.05±0.02(a)
D4	9.80±0.23(b)	6.27±0.37(a)	3.15±0.09(ab)	1.07±0.03(a)
D5	10.80±0.39(c)	6.03±0.23(a)	3.34±0.10(b)	1.08±0.04(a)

^α^ D3, D4, and D5 indicate the Meili wines produced from grapes at 1076 kg m^-3^, 1082 kg m^-3^, and 1089 kg m^-3^, respectively.

^β^ Values are the mean ± standard deviation (n = 3). Within columns, significant differences according to the Fisher’s LSD test at the *P* ≤ 0.05 level are indicated by different letters.

### Phenolic content in grapes and wines of different density classes

**Fig 2:**
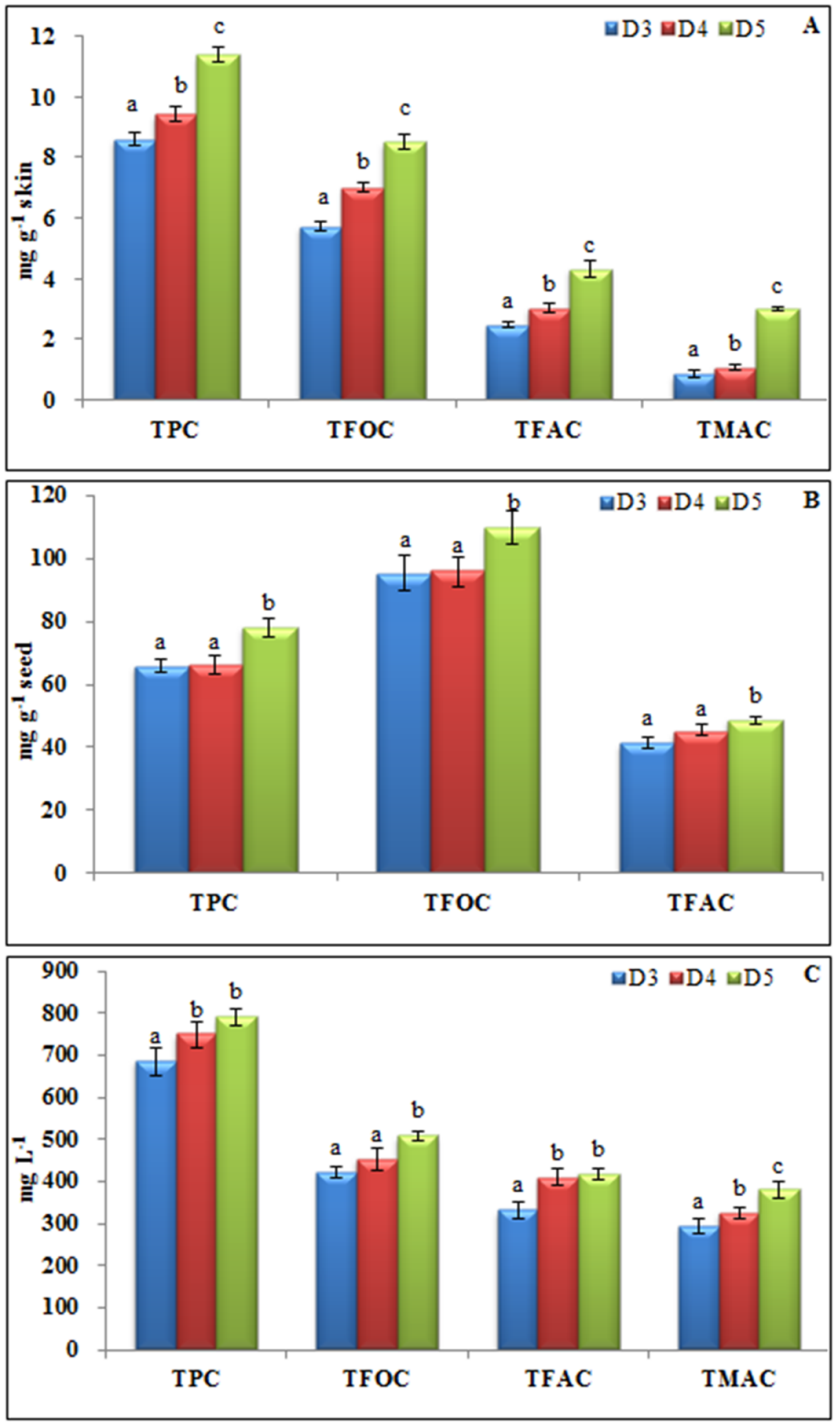
Phenolic content of Meili grapes and wine at different density levels. (A) Skin, (B) Seed, (C) Wine. D3 = 1076 kg m^-3^, D4 = 1082 kg m^-3^, D5 = 1089 kg m^-3^. TPC: total phenolic content expressed as gallic acid equivalent (GAE); TFOC: total flavonoid content expressed as rutin equivalent (RE); TFAC: total flavanol content expressed as (+)-catechin equivalents (CE); TMAC: total monomeric anthocyanin content expressed as cyanidin 3-glucoside equivalent (C3GE). Different letters within the same column indicate significant differences among the different density levels (LSD test; *P*≤0.05).

The TPC, TFOC, TFAC, and TMAC in the berry skins significantly increased with grape density (Fig 2A). The maximum phenolic content was determined for class D5, and the result confirmed the heterogeneity of Meili grapes at maturity and was in agreement with the findings of Torchio et al. [34] for Barbera grape. Changes in skin TPC, TFOC, and TFAC were found to increase gradually after veraison [13]. Variations in skin TMAC content must be carefully evaluated because it can directly influence the berry and final wine quality. Anthocyanin contents generally increase with soluble solids during ripening [17,20,22], and in the present study, the maximum anthocyanin content of 2.98 mg C3GE g^-1^ skin obtained for the D5 class was almost 3-fold higher than the lowest density for D3 (0. 85 mg C3GE g^-1^ skin). These results were consistent with the findings for Mencía grapes [22] and Muscat Hamburg grapes [35]. The variability in anthocyanin content was found be smaller under the Mediterranean climate characterized by a higher temperature and less rainfall [15]. The clusters could grow loosely with more homogeneous berries. However, environmental factors of sunlight, temperature, and air moisture to which berry skin is directly exposed varied more frequently under a continental monsoon climate. Additional climate impacts on phenolic compounds may influence the positive relationship between sunlight exposure and increased flavonol accumulation [36,37].

Differences in the phenolic compositions of the seeds within the three densities were poor compared to those found in the skin (Fig 2B). A significant increase in TPC, TFOC, and TFAC was only observed between density classes D4 and D5; the maximum contents were determined for the densest class (D5). No significant differences were found between D3 and D4 level. Although few studies have been reported on differences of TPC, TFOC, and TFAC in grape seeds at harvest, for Barbera grapes, significant variations in TFOC among three sugar content classes was found only in two experimental regions [34]. It appears that spatiality must be considered as responsible for variations in grape phenolics.

With regard to Meili wine, the phenolic content increased with grape density (Fig 2C). The highest value for TPC, TFOC, TFAC and TMAC was determined for the wine made from the D5 grapes. However, no significant variation in TPC and TFAC was found between the D4 and D5 classes. It is well known that the content of phenolic compounds in wine is highly related to the extractable phenolics in berry and maceration condition [38]. The textural properties of skin and seed had an active influence on phenolic extractability; for example the harder skins of Brachetto grapes allowed a greater release of pigments [39]. Moreover, different skin thickness and hardness were usually observed among grapes with different densities [39,40]. Hence, the different extractability of phenolics in berries might exist between D4 and D5 level. This phenomenon could further diminish the variance of TPC and TFAC content in wine between these two densities although the higher content of TPC and TFAC in skin and seed was determined for D5 as presented in Fig 2A and 2B.

### Antioxidant capacity of berries and wines of different density classes

The chemical diversity of antioxidants makes it difficult to separate and quantify from the grape and wine matrix. Therefore, the total antioxidant activity level was usually measured. However, a single antioxidant test for the examination of multifunctional antioxidants is insufficient, and more than one method were used to provide adequate information for the antioxidant properties of phenolics [41]. To characterize the antioxidant potential of Meili grape extracts and wines, three antioxidant assays of DPPH, CUPRAC, and HRSA were performed separately. The resulted data were presented in Table 3.

**Table 3:** Antioxidant capacity of Meili grapes and wines at different density levels.

Density level	DPPH			CUPRAC			HRSA		
	skin(μM TE g^-1^)^α^	seed(μM TE g^-1^)	wine(μM TE L^-1^)	skin(μM TE g^-1^)	seed(μM TE g^-1^)	wine(μM TE L^-1^)	skin(%)	seed(%)	wine(%)
D3	209.18±5.13(a)^β^	531.20±9.27(a)	4380±19(a)	78.36±5.20(a)	676.23±21.94(a)	5438±22(a)	24.61±3.46(a)	41.04±3.63(a)	45.85±4.65(a)
D4	210.09±5.01(a)	535.69±11.18(a)	4850±24(b)	90.79±4.61(a)	691.05±4.47(a)	5918±18(b)	27.65±3.55(b)	41.71±3.34(a)	52.24±4.83(b)
D5	225.51±5.31(b)	549.00±8.35(b)	5140±15(c)	114.96±6.68(b)	756.42±19.12(b)	7009±21(c)	34.35±3.69(c)	48.90±4.20(b)	59.59±5.4(c)

^α^ TE: trolox equivalents.

^β^ Values are the mean ± standard deviation (n = 3) and are based on the fresh weight of skin and seed, respectively. Within columns, significant differences according to the Fisher’s LSD test at the *P* ≤ 0.05 level are indicated by different letters.

DPPH, a stable 2,2-diphenyl-1-picrylhydrazyl radical was widely used to determine the free radical-scavenging capacity because the ethanolic extracts and wines were able to interact with the free DPPH radicals efficiently and quickly [42]. In terms of skin and seed fraction, DPPH radical scavenging capacity of Meili grapes increased significantly from D4 to D5 level although no increment was found between density D3 and D4 class. Moreover, the DPPH values of skin and seed extracts at any density level were higher than that of the common red cultivars registered in Turkey [43] and oriental *Vitis* species in China [44]; but they were lower compared to the wild grapes and hybrids native to Japan [45]. Antioxidant capacity of DPPH assay for Meili wines increased markedly with berry density, up to the highest value of 5140 μM TE L^-1^ at density D5. However, it was lower than that in Syrah and Cabernet Sauvignon wines and comparable with Pinot Noir wines [46]. These variances of antioxidant capacity might be related to the grape genotype that could have a deep impact on phenolic composition and antioxidant properties of grape extracts and wines.

Determination of the redox potential of a substance is a widely used method to estimate antioxidant capacity. CUPRAC assay focuses on detecting the reducing ability to reduction of Cu(II) to Cu(I) for the antioxidant [30]. The highest reducing power of CUPRAC assay was determined for skin and seed, respectively, in D5 level which was significantly higher than density D4. No significant differences were observed between density D3 and D4. The higher reducing potential ranging from 1321.5 to 1746.5 μM TE g^-1^ dry matter was measured for Cabernet Sauvignon powdered seeds with a small diameter [47]. The smaller powdered seeds were benefit for extraction of phenolic compounds that would contribute to the antioxidant capacity. The CUPRAC value of Meili wines increased obviously with berry density and reached to the maximum in D5 level as DPPH value showed.

Hydroxyl radicals, one of the most harmful free radicals generated as an oxygen byproduct could cause damage to proteins, lipids, and DNA which would lead to oxidative stress [48]. However, this damage could be effectively quenched by phenolics via scavenging of hydroxyl radicalse [49]. The hydroxyl radical-scavenging capacity of Meili grape extracts and wines increased significantly among the three densities except the HRSA value of seeds between D3 and D4 level. However, the average clearance rate of Meili skins was a little lower than Incrocio Prosperi grapes which showed higher total polyphenolic and anthocyanin index [50].

A positive correlation has been found between the phenolic composition and antioxidant capability of grapes [51]. In the present work, grape skin TPC, TFOC, and TMAC were also significantly and positively correlated with HRSA (r = 1.000, 1.000, 0.999, *P*≤0.05, respectively), similar to other studies [44,52]. TPC and TFOC values for the seed were also positively and significantly correlated with the clearance rate of hydroxyl radicals (r = 0.998, 1.000, *P*≤0.05, respectively). However, the positive correlations among phenolics content, DPPH, and CUPRAC were not obtained. It has been found that polyphenols showed different rankings of reactivity with different antioxidant assays [53]. Slight differences among these antioxidant values may be attributed to the multiple reaction characteristics and mechanisms of each method [54]. On the other hand, the antioxidant activity of phenolics did not depend exclusively on the total polyphenol contents [55] and were also determined by their molecular structure and more specially, by the position and degree of hydroxylation of the ring structure [56].

### Individual phenolic compounds of grapes and wines of different density classes

To further understand the phenolic profiles of Meili grapes and wines of different densities, phenolic compounds in the skin, seed, and wine were measured by HPLC. A dramatic increase was found from D3 to D4 for the sum of individual phenolic compounds in skin (Table 4). However, no difference with statistical significance was determined between the D4 and D5 levels. Correspondingly, the content of most of the individual phenolic compounds detected did not increase significantly among the three densities, except for salicylic acid and rutin. The difference in these two phenolics might be related to the higher UV-B exposure of the denser grapes because the biosynthesis of salicylic acid and rutin would be enhanced upon exposure to higher UV-B [57,58]. Salicylic acid was the predominant nonflavonoid monomer compound detected in Meili grape skin, with a concentration ranging from 13.12 to 15.81 μg g^-1^ skin. A significant increase in the rutin content was also determined between D3 and D4. Nevertheless, some individual flavonoid phenolics, such as (+)-catechin, (-)-epicatechin, quercetin, and kaempferol didn’t vary significantly among the three densities.

**Table 4:** Significant individual phenolic compounds in the skin of Meili grapes based on density classes (μg g^-1^ skin).

Phenolic compounds	Density level		
	D3	D4	D5
***Nonflavonoid phenolics***			
Hydroxybenzoic acids	27.61(40.21%)	33.52(42.79%)	34.90(44.18%)
Gallic acid	3.48 ± 0.08(a) ^α^	4.14 ± 0.10(b)	4.34 ± 0.06(b)
Benzoic acid	nd ^β^	nd	nd
Vanillic acid	4.41 ± 1.49(a)	5.38±0.23(a)	6.06 ± 0.87(a)
Syringic acid	6.16 ± 1.95(a)	8.11±0.66(a)	8.17 ± 0.93(a)
*p*-Coumaric acid	0.44 ± 0.12(a)	0.51±0.03(a)	0.52 ± 0.01(a)
Salicylic acid	13.12 ± 0.73(a)	15.38±0.79(b)	15.81±0.19(b)
Hydroxycinnamic acids	0.08(0.12%)	0.09(0.11%)	0.09(0.11%)
Chlorogenic acid	nd	nd	nd
Caffeic acid	0.08 ± 0.01(a)	0.09 ± 0.02(a)	0.09 ± 0.02(a)
Ferulic acid	nd	nd	nd
Stilbenes	1.48(2.16%)	1.63(2.08%)	1.75(2.22%)
*trans*-Resveratrol	1.48 ± 0.12(a)	1.63 ± 0.05(a)	1.75 ± 0.18(a)
Benzopyrone	13.73(19.99%)	14.05(17.93%)	13.84(17.52%)
Coumarin	13.73 ± 0.66(a)	14.05 ±0.88(a)	13.84 ± 0.89(a)
Sum of individual nonflavonoid phenolics	42.90(62.47%)	49.29(62.92%)	50.58(64.03%)
***Flavonoids***			
Flavan-3-ols	12.87(18.74%)	13.55(17.30%)	13.13(16.62%)
(+)-Catechin	10.59 ± 0.14(a)	11.04 ± 0.29(a)	10.13 ± 0.75(a)
(-)-Epicatechin	2.28 ± 0.59(a)	2.51 ± 0.32(a)	3.00 ± 0.44(a)
Flavonols	12.90(19.79%)	15.50(20.89%)	15.28(20.47%)
Rutin	1.74 ± 0.03(a)	3.76 ± 0.11(b)	4.23 ± 0.86(b)
Morin	nd	nd	nd
Quercetin	6.56 ± 0.11(a)	7.45 ± 1.25(a)	6.88 ± 0.60(a)
Kaempferol	4.60 ± 0.38(a)	4.29 ± 0.14(a)	4.17 ± 0.22(a)
Hesperetin	nd	nd	nd
Sum of individual flavonoid phenolics	25.77(37.53%)	29.05(37.08%)	28.41(35.97%)
Sum of individual phenolics	68.67 ± 2.54(a)	78.34 ± 1.77(b)	78.99 ± 2.95(b)

^α^ Values are the mean ± standard deviation (n = 3) and are based on the fresh weight of skin. Within lines, significant differences according to the Fisher’s LSD test at the *P*≤0.05 level are indicated by different letters.

^β^ nd: not detected.

The total content of individual phenolics in the seeds of Meili grapes increased dramatically with increasing grape density (Table 5). An increase of 61.24 μg g^-1^ seed and 39.79 μg g^-1^ seed of the sum of individual phenolics was measured between D3 and D4 and between D4 and D5, respectively. A significant increase was also determined for some important flavonoid compounds, such as (+)-catechin and (-)-epicatechin, between D3 and D4, with no increase between D4 and D5. A similar result was also found for salicylic acid in the seed. Kaempferol content in the seed declined with increasing grape density as in the skin. Hence, the ratio of quercetin derivatives (quercetin and rutin) to kaempferol in the seed and skin increased with density, respectively. Dihydroxy phenols (quercetin derivatives) are more potent antioxidants than monohydroxy phenols (kaempferol) and may increase as response to higher exposure to UV-B and temperature of denser grapes though temperature appeared to have a less significant influence [59]. The higher ratio (3.37) of quercetin derivatives to kaempferol in seeds of D5 level compared to that in density D3 and D4, 1.61 and 1.93, respectively, might contribute to the higher antioxidant activity of seed extracts as presented in Table 3.

**Table 5:** Significant individual phenolic compounds in the seed of Meili grapes based on density classes (μg g^-1^ seed).

Phenolic compounds	Density level		
	D3	D4	D5
***Nonflavonoid phenolics***			
Hydroxybenzoic acids	87.44(39.22%)	105.46(36.78%)	142.44(43.07%)
Gallic acid	24.32 ± 0.66(a) ^α^	26.81 ± 0.35(a)	31.07 ± 1.34(b)
Benzoic acid	4.13 ± 0.29(a)	6.26 ± 0.08(a)	16.61 ± 2.33(b)
Vanillic acid	38.54 ± 5.85(a)	41.92 ± 0.84(a)	63.70 ± 1.81(b)
Syringic acid	nd ^β^	nd	nd
*p*-Coumaric acid	5.50 ± 0.12(a)	9.81 ± 1.06(b)	6.65 ± 1.51(ab)
Salicylic acid	14.95 ± 1.89(a)	20.66 ± 1.88(b)	24.41 ± 0.71(b)
Hydroxycinnamic acids	42.15(18.90%)	46.00(16.04%)	53.23(16.09%)
Chlorogenic acid	nd	nd	nd
Caffeic acid	9.18 ± 0.05(a)	12.05 ± 0.83(a)	11.68 ± 1.43(a)
Ferulic acid	32.97 ± 1.06(a)	33.95 ± 2.96(a)	41.55 ± 2.11(b)
Stilbenes	0.93(0.42%)	1.10(0.38%)	1.37(0.41%)
*trans*-Resveratrol	0.93 ± 0.03(a)	1.10 ± 0.02(b)	1.37 ± 0.04(c)
Benzopyrone	5.95(2.67%)	17.34(6.05%)	14.83(4.48%)
Coumarin	5.95 ± 0.61(a)	17.34 ± 0.57(c)	14.83 ± 0.29(b)
Sum of individual nonflavonoid phenolics	136.47(61.21%)	169.90(59.26%)	211.87(64.06%)
***Flavonoids***			
Flavan-3-ols	60.01(26.91%)	88.63(30.91%)	92.36(27.92%)
(+)-Catechin	36.61 ± 2.03(a)	57.72 ± 7.76(b)	61.44 ± 7.57(b)
(-)-Epicatechin	23.40 ± 0.37(a)	30.91 ± 0.09(b)	30.92 ± 0.06(b)
Flavonols	26.49(11.88%)	28.17(9.83%)	26.52(8.02%)
Rutin	2.21 ± 0.43(a)	2.42 ± 0.78(a)	3.49 ± 0.25(a)
Morin	nd	nd	nd
Quercetin	14.14 ± 1.05(a)	16.15 ± 2.34(a)	16.96 ± 2.17(a)
Kaempferol	10.14 ± 1.60(b)	9.60 ± 0.70(b)	6.07 ± 0.03(a)
Hesperetin	nd	nd	nd
Sum of individual flavonoid phenolics	86.50(38.79%)	116.80(40.74%)	118.88(35.94%)
Sum of individual phenolics	222.97 ± 11.21(a)	286.70 ± 17.16(b)	330.75 ± 2.89(c)

^α^ Values are the mean ±standard deviation (n = 3) and are based on the fresh weight of seed. Within lines, significant differences according to the Fisher’s LSD test at the *P*≤0.05 level are indicated by different letters.

^β^ nd: not detected.

Obvious heterogeneity of individual phenolics content was also determined in Meili wines made from different density grapes (Table 6). Concerning the total content of individual phenolics, significant increment was determined among the three density classes. It increased by 26.38%, and 23.44% from D3 to D4 and from D4 to D5, respectively. The content of gallic acid, vanillic acid, salicylic acid, and ferulic acid mainly contributed to the sum of individual nonflavonoid phenolics at any density level. The concentration of these phenolic compounds was similar to the obtained level for the selected Chinese wines [60]. No significant change was measured for these phenolic acids between density D3 and D4 while a marked increase from D4 to D5 level was found for vanillic acid and ferulic acid. As for the flavonoids in Meili wines, the content of (+)-catechin, (-)-epicatechin and rutin comprised of the most content of total individual flavonoid phenolics. The content of (+)-catechin, one of the common subunit of proanthocyanidins increased significantly with grape density, up to a maximum of 53.26 mg L^-1^ at D5 level. Epicatechin, the epimer of catechin showed the same trend changing with grape density as in seeds. It increased markedly from density D3 to D4; but did not change with statistical variance between the two higher density levels. The content of rutin in wine of D5 was significantly higher than D3 and D4 wines in which no marked variance was determined. This discrepancy of variance for the different phenolic compounds among the three densities could be related to their contents in skin and seed. Moreover, the different extractabilities between these phenolics in the ethanolic matrix of wine during maceration may be another factor influencing their final content in wine [61].

**Table 6:** Significant individual phenolic compounds in the wine produced from Meili grapes based on density classes (mg L^-1^).

Phenolic compounds	Wine sample		
	D3 ^α^	D4	D5
***Nonflavonoid phenolics***			
Hydroxybenzoic acids	85.32 (38.29%)	104.40(37.08%)	137.01(39.41%)
Gallic acid	32.27 ±1.33(a) ^β^	36.25 ± 1.44(ab)	38.15 ± 1.29(b)
Benzoic acid	2.80 ± 0.49(a)	4.39 ± 0.53(a)	9.72 ± 1.04(b)
Vanillic acid	29.24 ± 1.63(a)	33.33 ± 2.24(a)	58.55 ± 1.00(b)
Syringic acid	5.03 ± 0.14(a)	8.20 ± 1.11(b)	8.03 ± 0.28(b)
*p*-Coumaric acid	3.97 ± 0.55(a)	8.04 ± 1.24(b)	7.85 ± 0.87(b)
Salicylic acid	12.02 ± 2.35(a)	14.20 ± 1.65(a)	14.72 ± 0.99(a)
Hydroxycinnamic acids	28.45(12.77%)	34.34(12.20%)	46.59(13.40%)
Chlorogenic acid	nd ^γ^	nd	nd
Caffeic acid	7.16 ± 0.50(a)	10.10 ± 1.53(ab)	11.80 ± 0.42(b)
Ferulic acid	21.29 ± 1.61(a)	24.24 ± 1.12(a)	34.79 ± 1.73(b)
Stilbenes	0.88(0.40%)	1.36(0.48%)	1.49(0.43%)
*trans*-Resveratrol	0.88 ± 0.10(a)	1.36 ± 0.21(a)	1.49 ± 0.26(a)
Benzopyrone	8.99(4.03%)	10.68(3.79%)	13.14(3.78%)
Coumarin	8.99 ± 0.75(a)	10.68 ± 0.62(ab)	13.14 ± 0.98(a)
Sum of individual nonflavonoid phenolics	123.64(55.49%)	150.78(53.54%)	198.23(57.03%)
***Flavonoids***			
Flavan-3-ols	64.53(28.96%)	88.82(31.54%)	97.43(28.03%)
(+)-Catechin	29.43 ± 1.71(a)	46.28 ± 1.51(b)	53.26 ± 1.53(c)
(-)-Epicatechin	35.09 ± 1.65(a)	42.55 ± 1.59(b)	44.17 ± 1.01(b)
Flavonols	34.64(15.55%)	41.99(14.91%)	51.95(14.94%)
Rutin	19.12 ± 1.99(a)	24.48 ± 1.60(a)	31.53 ± 1.66(b)
Morin	nd	nd	nd
Quercetin	9.71 ± 1.55(a)	11.67 ± 1.22(ab)	13.60 ± 0.30(b)
Kaempferol	5.81 ± 0.76(a)	5.84 ± 0.77(a)	6.81 ± 0.34(a)
Hesperetin	nd	nd	nd
Sum of individual flavonoid phenolics	99.17(44.51%)	130.81(46.46%)	149.37(42.97%)
Sum of individual phenolics	222.81 ± 3.20(a)	281.58 ± 6.25(b)	347.60 ± 7.64(c)

^α^ D3, D4, and D5 indicate Meili wine produced from grapes at 1076 kg m^-3^, 1082 kg m^-3^, and 1089 kg m^-3^, respectively.

^β^ Values are the mean ±standard deviation (n = 3). Within lines, significant differences according to the Fisher’s LSD test at the *P*≤0.05 level are indicated by different letters.

^γ^ nd: not detected.

**Fig 3:**
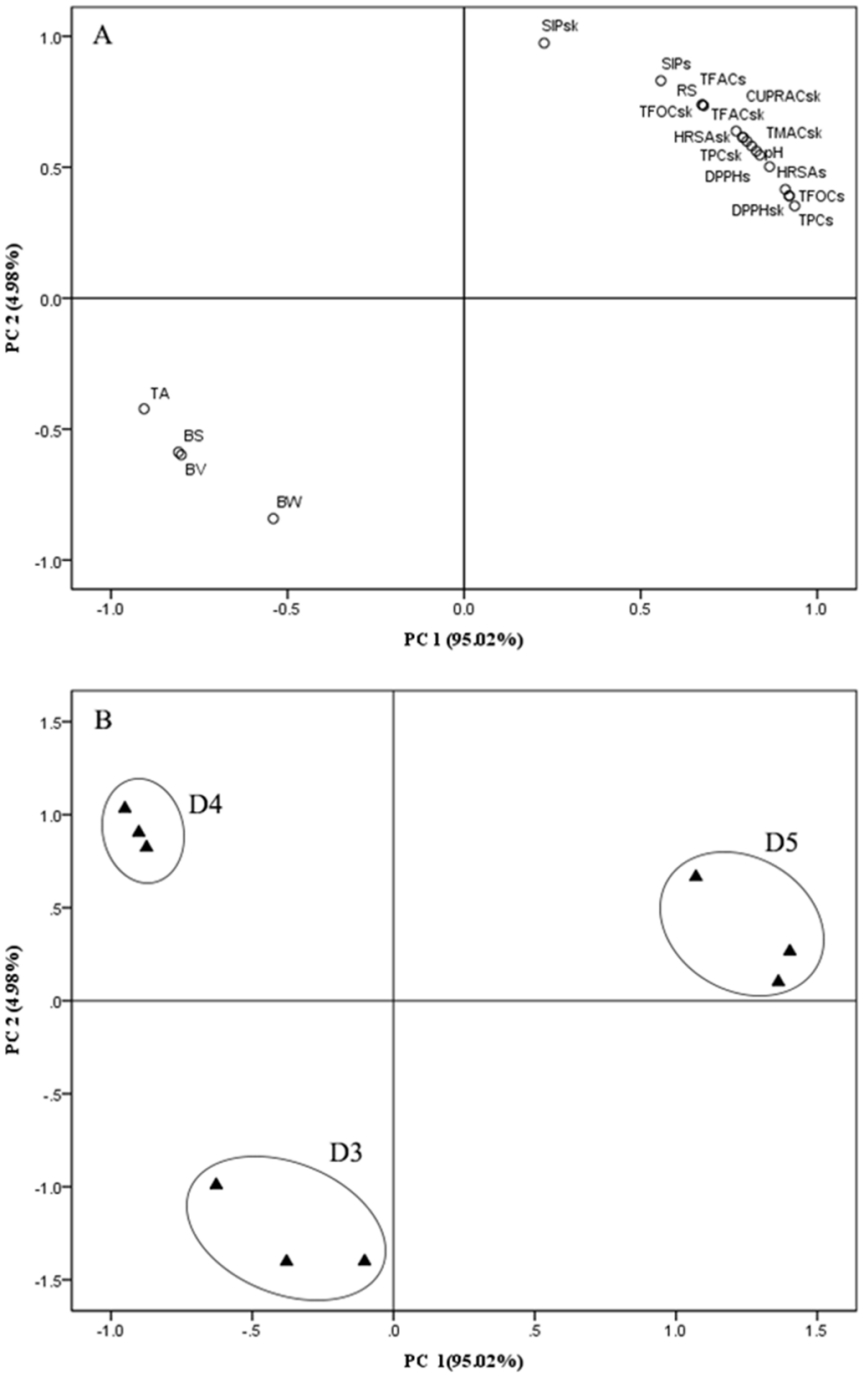
Output of principal component analysis (PCA) of Meili grapes at different densities. (A) Contribution of the individual variables to the PCA. (B) PCA. D3 = 1076 kg m^-3^, D4 = 1082 kg m^-3^, D5 = 1089 kg m^-3^. BW: berry weight; BS: berry surface; BV: berry volume; RS: reducing sugars; TA: total acidity; pH: pH value; TPCsk: total phenolic content in skins; TPCs: total phenolic content in seeds; TFOCsk: total flavonoid content in skins; TFOCs: total flavonoid content in seeds; TFACsk: total flavanol content in skins; TFACs: total flavanol content in seeds; TMACsk: total monomeric anthocyanin content in skins; SIPsk: sum of individual phenolic compound in skins; SIPs: sum of individual phenolic compound in seeds; DPPHsk: DPPH free radical-scavenging activity in skins; DPPHs: DPPH free radical-scavenging activity in seeds; CUPRACsk: cupric-reducing antioxidant capacity in skins; CUPRACs: cupric-reducing antioxidant capacity in seeds; HRSAsk: hydroxyl radical-scavenging activity in skins; HRSAs: hydroxyl radical-scavenging activity in seeds.

Principal component analysis (PCA) was performed to obtain a better understanding of the differences found among the berries of various densities (Fig 3). Two principal components were responsible for 100% of the variability in the original data. Component 1 was responsible for 95.02% of the cumulative variance and was mainly associated with the phenolic content and antioxidant capacity. Component 2 was responsible for 4.98% of the cumulative variance and was mainly associated with physical and technological maturity parameters. Three groups were clearly established and the densest grapes (D5) showed a higher phenolic content and antioxidant ability. However, the ratio of the D5 level at harvest was only 17.49% in the present study. Some types of viticulture management are expected to enhance the percentage of berries with high density under the continental monsoon climate.

## Conclusions

The present study demonstrated significant berry heterogeneity at harvest, which could result in differences in phenolic compounds and antioxidant capacity of Meili grapes and wine under a continental monsoon climate. The TPC, TFOC, TFAC, and TMAC contents in the skin and wine significantly increased with increasing grape density; the highest skin, seed, and wine phenolic compound contents and antioxidant capacities were found in the densest berries. The sum of individual phenolic compounds in the skin, seed, and wine increased with berry density, though no significant difference for the skin was observed between the two higher density classes. PCA demonstrated differences among the berries of the three density classes. The results of this study indicated that the chemical parameters of Meili grapes and wines are positively associated with the berry density at harvest under the continental monsoon climate.

## Supporting Information

S1 File The relevant data including physical and technological maturity parameters of Meili grapes, wine parameters, phenolics content and antioxidant activity of grapes and wines. (DOC)
