# Supplementary material for: Influence of Berry Heterogeneity on Phenolics and Antioxidant Activity of Grapes and Wines: A Primary Study of the New Winegrape Cultivar Meili (Vitis vinifera L.)
Source: PLoS One. 2016 Mar 14;11(3):e0151276. doi: 10.1371/journal.pone.0151276 (PMC4790946; doi:10.1371/journal.pone.0151276)
Supplement: S1 File — (DOC) [file pone.0151276.s001.doc]

S1 File

Supporting Information

**Table A. Physical maturity parameters of Meili grapes at harvest based on berry density classes.**

| Density level | Berry weight | Volume | Surface | Density level | Berry weight | Volume | Surface | Density level | Berry weight | Volume | Surface |
| --- | --- | --- | --- | --- | --- | --- | --- | --- | --- | --- | --- |
| D3 | 2.99 | 2.62 | 9.18 | D4 | 3.22 | 1.70 | 6.89 | D5 | 3.61 | 2.09 | 7.9 |
| D3 | 2.46 | 2.08 | 7.88 | D4 | 3.07 | 1.31 | 5.81 | D5 | 2.61 | 2.52 | 8.98 |
| D3 | 2.5 | 2.18 | 8.16 | D4 | 1.98 | 2.41 | 8.69 | D5 | 3.46 | 2.29 | 8.41 |
| D3 | 3.92 | 2.81 | 9.64 | D4 | 2.61 | 2.73 | 9.44 | D5 | 2.4 | 2.29 | 8.4 |
| D3 | 2.94 | 2.74 | 9.48 | D4 | 2.47 | 2.77 | 9.53 | D5 | 2.19 | 2.15 | 8.06 |
| D3 | 3.52 | 3.06 | 10.2 | D4 | 4.04 | 1.94 | 7.54 | D5 | 3.36 | 2.49 | 8.89 |
| D3 | 3.46 | 1.97 | 7.61 | D4 | 2.89 | 1.80 | 7.18 | D5 | 3.4 | 2.05 | 7.8 |
| D3 | 2.35 | 2.06 | 7.82 | D4 | 4.31 | 2.51 | 8.95 | D5 | 2.17 | 2.49 | 8.88 |
| D3 | 3.3 | 2.50 | 8.91 | D4 | 2.21 | 1.94 | 7.56 | D5 | 2.48 | 1.66 | 6.79 |
| D3 | 3.07 | 2.57 | 9.07 | D4 | 2.88 | 2.98 | 10.03 | D5 | 3.16 | 2.51 | 8.95 |
| D3 | 2.41 | 1.97 | 7.59 | D4 | 2.77 | 2.43 | 8.73 | D5 | 3.06 | 2.57 | 9.09 |
| D3 | 2.24 | 1.68 | 6.84 | D4 | 2.67 | 3.16 | 10.42 | D5 | 3.02 | 1.86 | 7.32 |
| D3 | 2.16 | 1.95 | 7.55 | D4 | 2.81 | 2.52 | 8.95 | D5 | 3.01 | 2.03 | 7.76 |
| D3 | 3.23 | 2.57 | 9.07 | D4 | 2.86 | 2.31 | 8.45 | D5 | 2.41 | 2.46 | 8.83 |
| D3 | 4.4 | 2.40 | 8.66 | D4 | 2.79 | 2.69 | 9.37 | D5 | 2.38 | 1.87 | 7.34 |
| D3 | 3.07 | 2.62 | 9.18 | D4 | 2.77 | 2.31 | 8.45 | D5 | 2.93 | 2.00 | 7.68 |
| D3 | 2.82 | 2.89 | 9.82 | D4 | 2.47 | 3.36 | 10.88 | D5 | 2.65 | 1.74 | 7.04 |
| D3 | 3.04 | 2.25 | 8.33 | D4 | 3.43 | 2.06 | 7.83 | D5 | 2.02 | 2.96 | 9.96 |
| D3 | 4.01 | 2.31 | 8.46 | D4 | 3.09 | 2.30 | 8.44 | D5 | 3.89 | 2.07 | 7.85 |
| D3 | 3.25 | 1.81 | 7.17 | D4 | 3.08 | 2.49 | 8.88 | D5 | 3.57 | 2.79 | 9.58 |
| D3 | 3.35 | 2.35 | 8.54 | D4 | 3.54 | 2.99 | 10.05 | D5 | 2.38 | 1.92 | 7.47 |
| D3 | 3.79 | 3.41 | 10.95 | D4 | 2.29 | 3.07 | 10.22 | D5 | 2.98 | 2.92 | 9.87 |
| D3 | 2.88 | 3.15 | 10.4 | D4 | 3.31 | 2.61 | 9.17 | D5 | 2.9 | 2.83 | 9.68 |
| D3 | 3.53 | 2.54 | 9 | D4 | 3.67 | 2.68 | 9.33 | D5 | 2.46 | 2.34 | 8.53 |
| D3 | 1.92 | 2.79 | 9.57 | D4 | 2.93 | 2.06 | 7.84 | D5 | 2.56 | 2.84 | 9.71 |
| D3 | 2.85 | 2.69 | 9.36 | D4 | 3 | 1.71 | 6.93 | D5 | 2.24 | 3.10 | 10.29 |
| D3 | 3.42 | 2.90 | 9.84 | D4 | 2.4 | 2.21 | 8.21 | D5 | 3.65 | 1.65 | 6.77 |
| D3 | 3.75 | 2.90 | 9.84 | D4 | 3.44 | 2.67 | 9.31 | D5 | 2.76 | 2.11 | 7.94 |
| D3 | 2.72 | 2.13 | 8 | D4 | 2.13 | 2.26 | 8.35 | D5 | 2.9 | 2.16 | 8.08 |
| D3 | 2.49 | 1.78 | 7.11 | D4 | 2.41 | 2.28 | 8.38 | D5 | 2.43 | 1.98 | 7.64 |

**Table B. Technological maturity parameters of Meili grapes at harvest based on berry density classes.**

| Density level | Reducing sugars | Total acidity | pH | Density level | Reducing sugars | Total acidity | pH | Density level | Reducing sugars | Total acidity | pH |
| --- | --- | --- | --- | --- | --- | --- | --- | --- | --- | --- | --- |
| D3 | 153 | 8.51 | 2.95 | D4 | 166 | 7.95 | 3 | D5 | 185 | 6.78 | 3.23 |
| D3 | 150 | 8.34 | 3.01 | D4 | 169 | 8.82 | 3.07 | D5 | 181 | 7.11 | 3.1 |
| D3 | 154 | 9.28 | 2.98 | D4 | 164 | 8.97 | 2.98 | D5 | 182 | 8.06 | 3.12 |

**Table C. Wine parameters.**

| Density level | Alcohol content | Titratable acidity | pH | Residual sugars |
| --- | --- | --- | --- | --- |
| D3 | 8.02 | 6.25 | 3.08 | 1.05 |
| D3 | 9.15 | 6.86 | 2.78 | 1.03 |
| D3 | 9.80 | 6.34 | 3.22 | 1.06 |
| D4 | 9.87 | 6.69 | 3.25 | 1.10 |
| D4 | 9.54 | 6.04 | 3.11 | 1.08 |
| D4 | 9.98 | 6.07 | 3.09 | 1.04 |
| D5 | 11.06 | 6.23 | 3.46 | 9.15 |
| D5 | 10.35 | 5.78 | 3.27 | 9.05 |
| D5 | 10.98 | 6.09 | 3.29 | 9.90 |

**Table D. P**henolics content in grapes and wines.

| Density level | TPCsk | TPCs | TPCw | TFOCsk | TFOCs | TFOCw | TMACsk | TMACw | TFACsk | TFACs | TFACw |
| --- | --- | --- | --- | --- | --- | --- | --- | --- | --- | --- | --- |
| D3 | 8.62 | 63.92 | 648 | 5.91 | 89.19 | 425 | 0.865 | 299 | 2.55 | 41.65 | 321 |
| D3 | 8.65 | 65.84 | 697 | 5.61 | 100.07 | 405 | 0.877 | 276 | 2.48 | 42.86 | 317 |
| D3 | 8.54 | 67.77 | 685 | 5.67 | 96.81 | 416 | 0.817 | 294 | 2.36 | 39.40 | 334 |
| D4 | 9.13 | 65.14 | 786 | 6.88 | 91.04 | 479 | 1.051 | 342 | 3.01 | 47.22 | 423 |
| D4 | 9.5 | 69.78 | 734 | 7.18 | 98.61 | 426 | 1.112 | 314 | 3.03 | 42.13 | 386 |
| D4 | 9.62 | 62.96 | 728 | 6.94 | 98.88 | 448 | 1.038 | 319 | 3.08 | 44.81 | 417 |
| D5 | 11.33 | 76.87 | 815 | 8.20 | 112.66 | 520 | 2.912 | 386 | 4.10 | 47.85 | 432 |
| D5 | 11.63 | 81.5 | 775 | 8.69 | 103.74 | 508 | 3.046 | 357 | 4.26 | 48.25 | 402 |
| D5 | 11.18 | 76.05 | 782 | 8.63 | 112.93 | 495 | 2.985 | 393 | 4.61 | 50.01 | 417 |

**Table E. Antioxidant activity of grapes and wines.**

| Density level | DPPHsk | DPPHs | DPPHw | CUPRACsk | CUPRACs | CUPRACw | HRSAsk | HRSAs | HRSAw |
| --- | --- | --- | --- | --- | --- | --- | --- | --- | --- |
| D3 | 207.79 | 527.18 | 4380 | 89.88 | 746.29 | 5416 | 24.13 | 40.8 | 50.24 |
| D3 | 211.63 | 535.69 | 4361 | 74.72 | 744.50 | 5439 | 25.04 | 41.76 | 41.00 |
| D3 | 208.11 | 530.73 | 4399 | 70.48 | 778.49 | 5459 | 24.67 | 40.56 | 46.30 |
| D4 | 211.70 | 542.78 | 4871 | 92.62 | 670.31 | 5936 | 28.05 | 41.34 | 57.24 |
| D4 | 207.84 | 531.44 | 4824 | 89.57 | 657.87 | 5918 | 27.88 | 42.01 | 47.60 |
| D4 | 210.73 | 532.86 | 4855 | 90.18 | 700.53 | 5900 | 27.03 | 41.78 | 51.89 |
| D5 | 224.34 | 546.40 | 5165 | 110.73 | 695.19 | 7021 | 33.67 | 48.98 | 53.70 |
| D5 | 224.02 | 547.82 | 5115 | 116.78 | 686.31 | 7022 | 35.05 | 49.05 | 60.74 |
| D5 | 228.17 | 552.78 | 5140 | 117.38 | 691.64 | 6985 | 34.34 | 48.67 | 64.32 |
